# A Rare Case of a Vocal Cord Foreign Body in an Infant: A Case Report

**DOI:** 10.7759/cureus.29213

**Published:** 2022-09-15

**Authors:** Deemah H Bukhari, Abdulrahman F Kabli, Taghreed S Alharthi, Esraa Sendi, Atef A Rashed

**Affiliations:** 1 Otolaryngology - Head and Neck Surgery, Maternity and Children’s Hospital, Makkah, SAU; 2 Medicine, Umm Al-Qura University, Makkah, SAU; 3 General Practice, Maternity and Children’s Hospital, Makkah, SAU; 4 Pediatrics, Maternity and Children’s Hospital, Makkah, SAU

**Keywords:** sharp object, croup, glass, infant, foreign body

## Abstract

A foreign body (FB) is an object or item that is foreign to the area in which it is found. FB in the airway, accompanied by the esophagus, is a common overnight emergency in pediatric otolaryngology. Here we report a case of a healthy 11-month-old girl who presented in the emergency room with stridor and a weak cry. The patient was admitted as a case of croup (laryngotracheobronchitis) and treated with multiple antibiotics for more than five days but showed no improvement, then consulted the ear, nose, and throat team (ENT).

## Introduction

A foreign body (FB) refers to an object or item considered foreign to the area in which it is discovered [1]. In pediatric otolaryngology, FB in the airway, accompanied by the esophagus, is indeed a frequent overnight emergency [2]. FB will often be aspirated into the tracheobronchial tree, leading to a variety of respiratory manifestations, including acute respiratory distress and chronic lung disease [3]. The majority of patients are below the age of four [3]. Aerodigestive tract FBs are a major contributor to mortality and morbidity in the pediatric age group since they are naturally driven to explore their surroundings via oral exploration [4]. There are limited reports in the literature regarding laryngeal FBs. It commonly presents with acute airway obstruction [5]. Airway obstruction might be partial or complete. When the upper airway is partially blocked or if the obstruction is distal to the carina, this is referred to as partial obstruction [3]. Death may occur if the FB is not coughed out or aspirated further into the lower airway [5]. Commonly, it may lodge in the bronchus (83%) followed by the trachea (12%), larynx (2-9%), or hypopharynx (5%) [6]. Patients may present within weeks to months following FB aspiration. Typically, FB settles in the periphery, distant to the larynx or trachea. Besides, FB with a sharp or irregular shape remains trapped in the larynx or trachea [6]. We report a case of a healthy 11-month-old girl who went to the emergency room complaining of cough and shortness of breath due to the ingestion of a foreign object without fever.

## Case presentation

**Figure 1:**
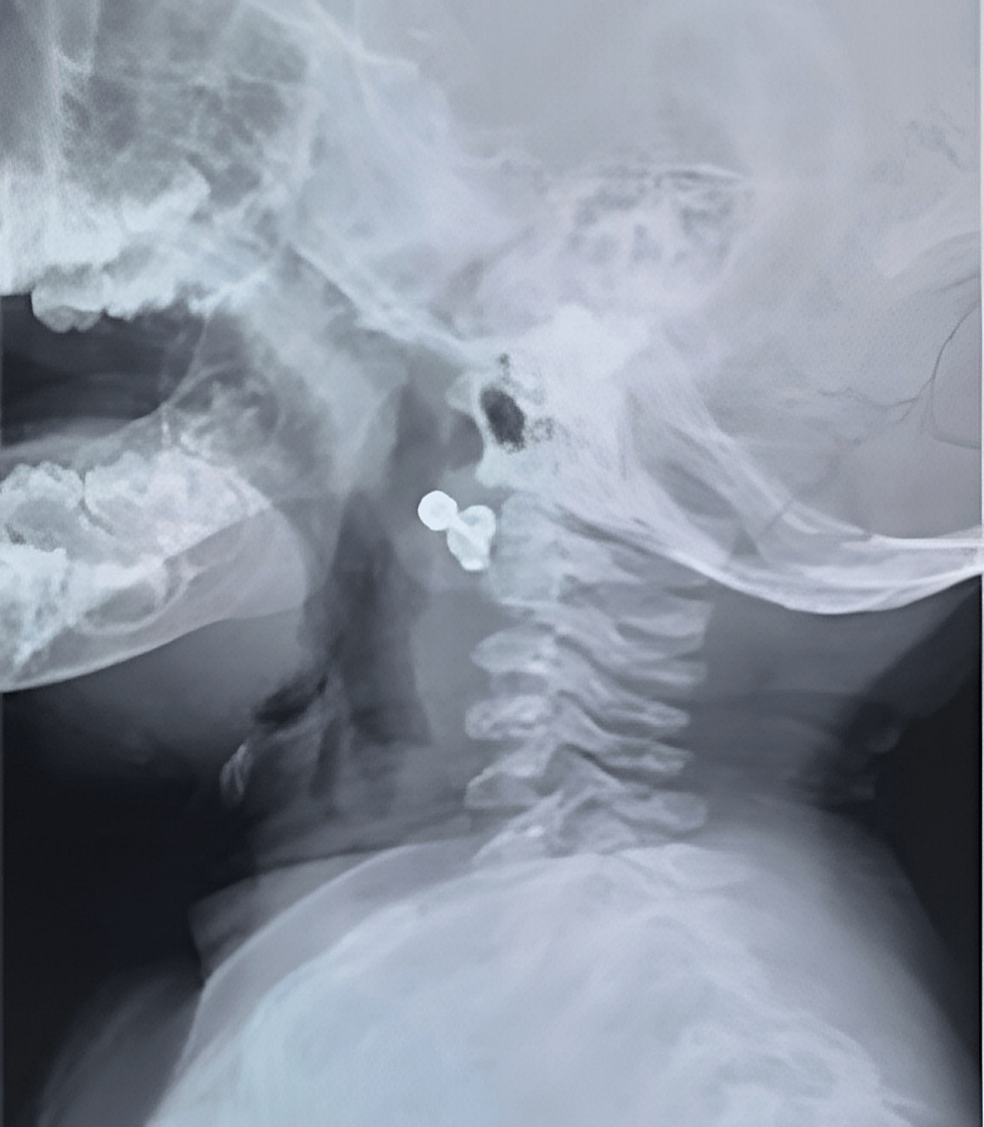
Lateral view neck X-ray

A healthy 11-month-old girl presented in the emergency room with stridor and a weak cry. The patient was admitted as a case of croup (laryngotracheobronchitis) and treated with multiple antibiotics for more than five days but showed no improvement, then consulted the ENT team. When we saw the patient, the history of FB aspiration was unsure by the parents, but there was a sudden history of stridor and a weak cry. On examination, the patient was physically healthy, vitally stable, afebrile, conscious and active with good oral intake. No previous medical or surgical history. The complete blood count (CBC), electrolytes, and coagulation profile that we ordered for her were all within normal range. A lateral neck X-ray was normal (Figure 1).

**Figure 2:**
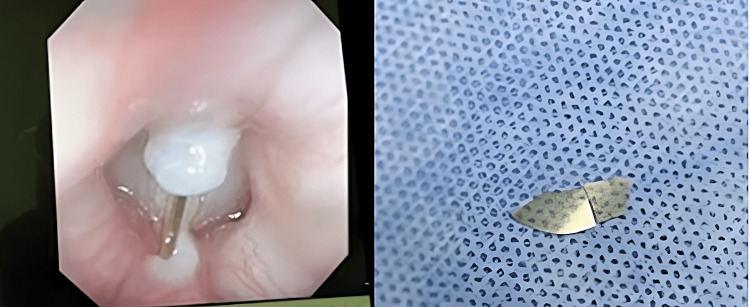
Bronchoscope view of the piece of the glass

We decided to do a fiberoptic scope on the patient and accidentally found a foreign body glass between the vocal cord and planned to remove it in the operating room by bronchoscope. Intraoperatively by bronchoscope, we saw a piece of glass trapped between the vocal cords, with no damage to vocal cords or surrounding structures. There was evidence of mild reactive granulation tissue at the site of foreign body impatience (Figure 2).

We successfully and carefully remove it without incident. The patient improved immediately, and the voice returned to normal; no more stridor, the patient was discharged home. Furthermore, during follow-up, the patient appeared healthy and active without any complaints.

## Discussion

Aspiration of FB, one of the causes of respiratory distress in infants, is a dangerous and potentially life-threatening condition.

FB aspiration is challenging to diagnose due to its clinical manifestations. Symptoms differ based on the degree of airway obstruction or the location of the FB. The most frequent sites of FB aspiration in children are the right bronchi (60%) and left bronchi (23%), followed by the trachea/carina (13%), larynx (3%) and bilateral (2%) sites [6].

Most FB aspiration cases are between one and five years old when children are naturally inquisitive and attempting to interact with their environment. Most cases of FB aspirations in infants younger than one year result from an older sibling playing with the infant and attempting to feed it. Children with foreign body ingestion or aspiration may exhibit a wide range of symptoms; the condition is sometimes misdiagnosed, and the patient may be left unattended for extended periods. Many laryngeal bodies pass into the bronchus or are coughed out, so they are rarely reported or suspected [7]. Such laryngeal FBs can occasionally lead to respiratory obstruction and reflex laryngospasm [2].

As was the case here, two children were playing together until one forced the other to ingest a pen cap, a common occurrence among preschool children. They were surprised that the child had no symptoms, and a bronchoscope was used to remove the pen cap that was found below the vocal cord after a computed tomography (CT) [8]. Alternatively, it may cause multiple symptoms or mimic other diseases, making diagnosis difficult. Following the case of a one-year-old child who complained of fever and cough and was treated with multiple antibiotics as a case of bronchopneumonia but showed no improvement, it was determined that the child did not have bronchopneumonia. They ordered a chest X-ray and discovered hyperinflation and hyper-translucency in the left lung, which obstruction may have caused. A bronchoscopy was done, which revealed a long plastic piece [9]. In Swibel Rosenthal et al. a healthy child was diagnosed with croup and given albuterol, nebulized racemic epinephrine until the family mentioned a choking incident involving a rubber hand before nine months. In the past nine months, she had multiple visits to the emergency room for the same condition. A flexible fibre optic laryngoscopy (FFL) was performed through her nose as the likelihood of a foreign body aspiration increased. Using a bronchoscope, a visible loop of rubber band was removed from the supraglottis [10]. Previous cases describe the variety of how foreign body aspiration can present with symptoms or asymptomatic, which makes establishing the diagnosis a little difficult. In our case, it is uncommon for a piece of glass with a sharp end to remain between the vocal cords without harming other structures. In addition, a detailed medical history from the child's parents or the child's own concerns, if they can speak, are essential for providing the correct medical care. We aim to report this case to describe the rare presence of sharp glass within vocal cords and extract without any injury and, to increase awareness of physicians toward various clinical manifestations of FB aspiration.

## Conclusions

Foreign body ingestion can present with different clinical manifestations, which sometimes makes it difficult. We aimed to increase the awareness of different presentations of foreign body aspiration. Every physician should consider it in their differential and take a good history from the parents when there is suspicion.

## References

[1] Mohanty S, Behera IC (2014). An unsafe foreign body at an unsafe site of airway. Int J Phonosurg Laryngol.

[2] Sharma VK, Rana AK, Sharma R (20191). Uncommon and dangerous foreign body in an infant’s larynx: an interesting presentation. Otorhinolaryngol Clin.

[3] Mittal A, Bhargava R, Kumar S (2013). Glottic foreign bodies in infants: a series of four cases. Case Rep Clin Med.

[4] Miller RS, Willging JP, Rutter MJ, Rookkapan K (2004). Chronic esophageal foreign bodies in pediatric patients: a retrospective review. Int J Pediatr Otorhinolaryngol.

[5] Karakoç F, Karadağ B, Akbenlioğlu C, Ersu R, Yildizeli B, Yüksel M, Dağli E (2002). Foreign body aspiration: what is the outcome?. Pediatr Pulmonol.

[6] Fidkowski CW, Zheng H, Firth PG (2010). The anesthetic considerations of tracheobronchial foreign bodies in children: a literature review of 12,979 cases. Anesth Analg.

[7] Hewlett JC, Rickman OB, Lentz RJ, Prakash UB, Maldonado F (2017). Foreign body aspiration in adult airways: therapeutic approach. J Thorac Dis.

[8] Jain S, Kashikar S, Deshmukh P, Gosavi S, Kaushal A (2013). Impacted laryngeal foreign body in a child: a diagnostic and therapeutic challenge. Ann Med Health Sci Res.

[9] Roy K, Amin SK, Setu M (2017). An unusual case of foreign body aspiration: a case report. Anwer Khan Mod Med Coll J.

[10] Swibel Rosenthal LH, Smith-Bronstein V, Cervantes S, Schroeder JW Jr (2018). A chronic glottic foreign body diagnosed by radiograph after 9 months of symptoms. Case Rep Pediatr.

